# Stochastic-Biomechanic Modeling and Recognition of Human Movement Primitives, in Industry, Using Wearables

**DOI:** 10.3390/s21072497

**Published:** 2021-04-03

**Authors:** Brenda Elizabeth Olivas-Padilla, Sotiris Manitsaris, Dimitrios Menychtas, Alina Glushkova

**Affiliations:** Centre for Robotics, MINES ParisTech, PSL Université, 75006 Paris, France; sotiris.manitsaris@mines-paristech.fr (S.M.); dimitrios.menychtas@mines-paristech.fr (D.M.); alina.glushkova@mines-paristech.fr (A.G.)

**Keywords:** movement modeling, state-space representation, gesture recognition, wearable sensors, ergonomics

## Abstract

In industry, ergonomists apply heuristic methods to determine workers’ exposure to ergonomic risks; however, current methods are limited to evaluating postures or measuring the duration and frequency of professional tasks. The work described here aims to deepen ergonomic analysis by using joint angles computed from inertial sensors to model the dynamics of professional movements and the collaboration between joints. This work is based on the hypothesis that with these models, it is possible to forecast workers’ posture and identify the joints contributing to the motion, which can later be used for ergonomic risk prevention. The modeling was based on the Gesture Operational Model, which uses autoregressive models to learn the dynamics of the joints by assuming associations between them. Euler angles were used for training to avoid forecasting errors such as bone stretching and invalid skeleton configurations, which commonly occur with models trained with joint positions. The statistical significance of the assumptions of each model was computed to determine the joints most involved in the movements. The forecasting performance of the models was evaluated, and the selection of joints was validated, by achieving a high gesture recognition performance. Finally, a sensitivity analysis was conducted to investigate the response of the system to disturbances and their effect on the posture.

## 1. Introduction

To fulfill market demands within specific time limits, job specifications and budget restrictions, the tasks performed by manual laborers in the industrial sector are becoming more challenging and complex. The tasks demanded of them require workers to go sometimes beyond their natural physical limitations, performing repetitive tasks for long periods of time. Being subjected to such constant physical strain leads to work-related musculoskeletal disorders (WMSDs) [1]. WMSDs can cause permanent or temporary damage to tissue, such as muscles, bones, joints or tendons, caused by cumulative microdamage, where the internal tolerance of the tissues is eventually exceeded. WMSDs are the most common work-related health issue in Europe [2], entailing consequences for workers and for the companies that employ them, that have to contend with high levels of sick leave and drops in productivity.

The ability to record accurate measurements for ergonomic analysis is essential as it provides ergonomists with quantitative measures of workers’ performance. This represents an added value in preventing ergonomic risk. Risk factors such as assuming awkward postures and performing highly repetitive or physically demanding tasks are often associated with WMSDs [2], mostly when occurring at high levels of repetition or in some kind of combination. Several rules and methods were established to identify ergonomic risks that workers might be exposed to during their professional activities. Three different measurements were used for these evaluations [3]. The first was self-assessment, where workers were asked to fill out a questionnaire indicating their level of exposure to diverse risk factors, including how tired they felt after their shift or if they had assumed any dangerous postures during their tasks. The second measurement is through observation by others, where an ergonomist observes the workers during their shift and completes a heuristic evaluation based on standards that indicate human physical limitations and abilities (e.g., the ISO 11226:2000 and EN 1005-4). These standards are mostly based on the deviation of the working posture from the neutral pose. The higher the deviation, the higher the risk of developing WMSD. Some existing questionnaries that use this approach are the Rapid Upper Limb Assessment (RULA) [4], Ergonomic Assessment Worksheet (EAWS) [5], and Ovako Working Posture Analysing System (OWAS) [6]. The third technique consists of direct measurement and primarily involves implementing a biomechanical-based analysis, where the loads and external forces the workers are exposed to are considered in the evaluation. An example of a direct measurement method is the National Institute of Occupational Safety and Health (NIOSH) lifting equation [7], which helps assess whether lifting a load is acceptable. Another is the Liberty Mutual manual materials handling tables [8], which indicate the load range that certain male or female members of the population may be able to lift, lower, carry, push or pull as part of their daily work, without the risk of developing WMSDs.

While methods based on self-assessment or visual observation, are quick and straightforward ways to evaluate, they are not always accurate and precise. They are quite subjective, since they are dependant on the worker’s feelings or sensations, or on the powers of observation of the ergonomist, leading, quite possibly, to low accuracy and high intra- and inter-observer variability [9]. For methods based on direct measurements, laboratory equipment is usually required, such as optical motion capture systems and force plates to measure external forces. This equipment requires a large infrastructure and is thus rather impractical and difficult to use in the workplace. Moreover, using these technologies involves bringing workers to the laboratory, causing inaccurate measures since they lack authenticity and are not real workplace scenarios. Recent research has started to develop alternative sensor-based automated evaluation methods, using cameras or body-mounted inertial sensors [10,11,12,13]; however, ergonomic evaluation in these studies relies purely on joint angle thresholds, which can only identify risks related to static postures [4,5,6].

The work presented in this paper aims to further expand the scope of the analysis conducted in current ergonomic evaluations by modeling professional movements. The hypothesis formulated here is that by modeling the workers’ dynamics, it is possible to extract information about the contribution of body joint movements to various ergonomic risks. Moreover, with the learned models, it is possible to predict the motion trajectory of body joints and thus detect any possible future exposure to postural ergonomic risk.

For the purposes of this research, human motion modeling and trajectory predictions were made using a Gesture Operational Model (GOM) [14], which consists of a system of equations based on different assumptions about the dynamic relationship of body parts. The methodology was validated by evaluating the forecasting performance of the system and by improving the recognition performance of professional movements, using four datasets. The first and second datasets were taken from professional movements executed in factories concerned with television production and airplane manufacturing, respectively. The third dataset was composed of gestures performed in a glassblowing workshop, while the fourth dataset of motion primitives, with different ergonomic risk levels, according to EAWS [5], was recorded in a laboratory.

In Section 2, which follows, the present state-of-the-art related to motion analysis for modeling, prediction, and pattern recognition, will be presented, while the methodology and evaluation procedures we used are described in Section 3. Section 4 presents the results of the experiments conducted on the four datasets, Section 5 discusses our findings and results, followed by the presentation of our conclusions in Section 6.

## 2. State-of-the-Art

### 2.1. Motion Analysis Based on Body Structure

In the past, biomechanical, stochastic, and hybrid models have been used to represent human motion and these models were then used to study the coordinated mechanical interaction between bones, muscles, and joints within the musculoskeletal system. The modeling of human movements, and their changes, caused by internal and external action forces has generally been addressed with biomechanical models. These models represent the human body as a set of articulated links in a kinetic chain where joint torques and forces are calculated using anthropometric, postural, and hand load data [15]. Inertial data, such as accelerations and velocities, and information about external forces like ground reaction forces from force plates, are used as input for biomechanical models [16]. When dealing with inverse dynamics, quantitative information about the mechanics of the musculoskeletal system, while performing a motor task, is extracted. Most previous studies have used biomechanical modeling to extract the kinematic and kinetic contributions of the joints, in diverse motor tasks, then investigate the mechanical loading of the joints and their response to ergonomic interventions. To analyze the ergonomic impact of different postures on human joints, Menychtas et al. [17] applied the Newton–Euler algorithm for the computation of upper body joint torques. The normalized integral of joint angles and joint torques was then calculated to describe the kinematic and kinetic contribution of the body joints when awkward poses are assumed. The method identified which joints moved the most during the tasks and were under the most strain while performing ergonomically dangerous gestures. Faber [18] used a spanned inverse dynamics model to estimate 3D L5/S1 moments and ground forces, then compared symmetric, asymmetric, and fast trunk bending movements through ergonomic analysis. Similarly, Shojaei [19] estimated the reaction forces and moments of the lower back, in manual material handling (MMH) tasks, to assess age-related differences in trunk kinematics and mechanical demands on the lower back.

In previous research, statistical modeling has been used to learn the stochastic behavior of human motion. These models capture the variance information of body motion trajectories and have been used both to estimate human intentions and label human activities. In order to infer intentions from observed human movements in real-time, Wang [20] presented the Intention-Driven Dynamics Model (IDDM), based on Gaussian processes. The dynamics model assumes that the goal directs human action, meaning that the dynamics change when the actions are based on different intentions. The study proved that including human dynamics in the modeling benefits the prediction of human intentions. In order to capture the motion patterns that emerge in typical human activities (e.g., walking and running), Argwal [21] trained a mixture of Gaussian auto-regressive processes with joint angles and position trajectories. The dynamic models take advantage of local correlations between joints motion to track complicated movements successfully (turns in different directions) using only 2D body measures (joint positions and joint angles). To segment and analyze human behaviours, Devanne [22] applied a Dynamic Naive Bayes model to capture the dynamics of elementary motions and to segment continuously in long sequences diverse human behaviors.

Hybrid methodologies that take into consideration human biomechanical structure and the stochastics of motion have been developed to improve the analysis of the random outcomes of movement. A hybrid model, designed to predict the probability of injury and identify factors contributing to the risk of non-contact anterior cruciate ligament (ACL) injuries, has been proposed by Lin [23]. A biomechanical model of the ACL estimated the lower leg kinematics and kinetics. In turn, the means and standard deviations of the number of simulated non-contact ACL injuries, injury rate, and female-to-male injury rate were calculated in Monte Carlo simulations of non-contact ACL injury and non-injury trials. *T*-tests revealed the biomechanical characteristics of the simulated injury trials. Donnell [24] used a two-state Markov chain model to represent the survival of surgical repair from rotator cuff. The load applied to the shoulder and the structural capacity of tissue were the random variables. The analysis was based on the application of structural reliability modeling. By introducing this new modeling paradigm for explaining clinical retear data, the model successfully predicted the probability of rotator cuff repair retears and contributed to understanding their causes. To describe the cooperation of body parts in the execution of professional movements, Manitsaris [14] proposed the Gesture Operational Model (GOM), based on state-space modeling. GOM offered insights into the dynamic relationship between body parts, within the execution of a movement, according to the statistical significance of its various assumptions and their dependencies on the motion of other body parts.

### 2.2. Motion Trajectory Prediction

The problem of human motion trajectory prediction has been researched extensively in the past. There is a growing interest, in the industrial sector, in implementing systems that allow prediction of how workers’ motion descriptors will unfold over time, and to incorporate this knowledge in a pro-active manner e.g., to facilitate human-robot collaboration or risk prevention. There are three prediction approaches, which are based on how human motion is represented and how the behavior pattern is formulated. Physics-based models are explicitly defined dynamic models explicitly defined and follow Newton’s Law of Motion. Pattern-based models, on the other hand, learn statistical behavioral patterns that emerge, based on the observed motion trajectories. Plan-based models are concerned with reasoning about the intention behind the movement and the goal of the performer.

#### 2.2.1. Physics-Based Models

Physics-based models predict future human motions according to a defined dynamic model (*f*). This model follows the form of a state-space representation:

(1) $$s\left(t+1\right)=f\left(s(t),u(t),t\right)+w\left(t\right)$$

where s(t+1) is the prediction, s(t) is the current motion state of the system, u(t) is the input, and w(t) the process noise. The motion is predicted by forward simulating the dynamic equations that follow the physics-based model. Physics-based models have tended to use kinematic models for prediction and these represented the motion states as position, orientation, velocity, or acceleration and linked the observations to the state’s evolution. Some examples of kinematic models used are constant velocity (CV) [25], constant acceleration (CA) [26], and coordinated turn (CT) [27]. These models describe the agent’s motion based on the mathematical relationship between the movement parameters (e.g., position, velocity, acceleration) without considering the external forces that affect the motion. Kinematic models are frequently used for prediction due to their simplicity and acceptable performance, under the conditions of little motion uncertainty, or short-term prediction.

For the prediction of pedestrians’ position trajectories, previous studies have applied Kalman Filters (KFs), with kinematic models such as CV and CA [26,28]. The main application of KF is for tracking the pedestrian position according to the estimated velocity or acceleration. Zernetsch [29] applied a kinematic model for trajectory prediction of cyclists that consisted of a CV model for the computation of all significant forces, such as the driving force and resisting force, composed of acceleration resistance, rolling resistance, and air resistance. In order to determine the kinematic model parameters, a curve-fitting approach was used, with motion profiles of cyclists that were recorded with a video camera and laser scanners at a public intersection.

For the prediction of movements with a high level of uncertainty, previous studies have used multi-model (MM) methods. These methods fuse different motion modes (e.g., sudden accelerations, linear movements, maneuvers) to describe complex motions (e.g., pedestrians or vehicles in public areas), where a dynamic model represents each mode. Pool [30] applied an MM approach to predict cyclists’ motion based on their motion strategies (go straight, turn left or right $45^{\circ }$ or $90^{\circ }$). Whenever a strategy does not comply with the road topology, the probability of the strategy is set to zero, in place of prediction. A multi-model approach for pedestrian trajectory prediction has been presented by Kooij [31], which uses Switching Linear Dynamical Systems (SLDS) to model maneuvering pedestrians that shift between motion models (e.g., walking, stopping). Then, a Dynamic Bayesian Network (DBN) predicts the pedestrian movements based on the SLDS model. The latent variables consisted of the pedestrian location, curb location and head orientation (indicating awareness of oncoming vehicles). The results proved that including context cues in the analysis improves overall prediction accuracy. Manitsaris [14] adequately addressed the forecasting trajectories of a 3D skeleton’s joint positions by using state-space modeling. The state variables corresponded with the dynamic association of body parts, their synergies, their serial and non-serial mediations, and the two previous positions of the body part represented. This study, by including information about other body parts in the representation of each body part, boosted the forecasting performance of the system due to the strong dynamic relationship between them.

Physics-based approaches are appropriate, where an explicit transition function can be defined for modeling the agent’s motion dynamics, as well as the influence of other agents and of their surroundings on it. The main drawback of using physics-based approaches is that they do not perform well for very complex situations (e.g., public areas with multiple agents). Moreover, their use is commonly limited to short-term predictions and obstacle-free environments.

#### 2.2.2. Pattern-Based Models

Pattern-based approaches, unlike physics-based approaches, learn human motion behaviors by fitting models to data. For the prediction of pedestrian trajectories, Quintero [32] presented the Gaussian process dynamical models (B-GPDMs). The system can reduce the 3-D time-related information extracted from key positions on the pedestrians’ bodies into only two observations, used for the prediction. The most similar model to the multiple models of four activity types (e.g., walking, stopping, starting and standing) is then selected to estimate future pedestrian states. For the motion prediction of multiple people, Kucner [33] used Gaussian Processes and their mixtures to model multimodal distributions, representing speed and orientation in joint space, for the purpose of modeling the motion of people and mapping their flow in the area analyzed.

Neural Networks have achieved promising performances for time-series prediction [34,35,36]. Among the most popular are the Long Short-Term Memory (LSTM) networks to predict human [34,35] and vehicle motion [36]. For the trajectory prediction of pedestrians’ 2D position and orientation, Sun [34] incorporated spatial and temporal context information into an LSTM to learn the human activity patterns generated in different environments at different times of the day. Xue [35] proposed the Social-Scene-LSTM (SS-LSTM), which uses three LSTMs to capture person, social and scene scale information. In turn, the output of the three networks is used by an LSTM decoder for the prediction of pedestrian trajectory coordinates. Srikanth [36] has proposed a robust model for future trajectory prediction of vehicles, where a simple Encoder-Decoder model connected by a convolutional LSTM was used to learn vehicle temporal dynamics, including semantic images, depth information and other vehicles’ positions. In this study, the use of scene semantics improved the prediction performance over models that only use information such as raw pixel intensities or depth information.

For the capturing of more complex unknown dynamics, it has to be admitted that pattern-based approaches have outperformed physics-based approaches; however, they require a large amount of data to train the model to avoid generalization issues. To improve the prediction performance, pattern-based and physics-based approaches have benefited from integrating context information into their observations. The studies that included information about the shape and structure of the environment, together with the external forces that the person or object is exposed to, or information about their interaction with other agents (e.g., people, vehicles or robots) produce more precise predictions in numerous cases [37].

#### 2.2.3. Planning-Based Models

The third prediction approach employs Planning-based models. Unlike the previous approaches, these assume rationality, in the case of tracked human movements and their long-term motion goals. This approach computes path hypotheses that allow the agent to reach their motion goals by considering the impact of current actions on future motions. The prediction is made using a predefined cost function, based on intended motion goals or inferred cost function, according to the observed trajectories. Best and Fitch [38] have proposed a Bayesian framework to estimate pedestrians’ intended goal destination and future trajectory. The framework is based on multimodal hypotheses of the intended goal, and the long-term trajectory that decreases the distance to the intended goal is selected. By seeing the trajectory prediction as an optimization problem, Lee [39] suggests a deep stochastic Recurrent Neural Network (RNN) Encoder-Decoder framework for trajectory prediction of multiple vehicles in complex scenes. The model obtains a diverse set of hypothetical trajectories which takes into consideration the agent interactions, scene semantics, and expected reward function. The single end-to-end RNN encoder-decoder network captures the past trajectories and incorporates the information into the inference process to improve prediction accuracy.

In order to use planning-based approaches, the goals that the agents under analysis are trying to achieve must first be explicitly defined, and the context information about the environment surrounding the agent must be provided for the model. Planning-based approaches usually perform better for long-term predictions than do physics-based approaches and also tend to have less generalization issues than Pattern-based approaches. The downside of these approaches is that as the complexity of the prediction problem increases (e.g., long-term predictions, multiple agents and size of the environment), so does the running time for training the models.

### 2.3. Human Gesture Recognition

Ergonomic evaluations have been conducted by identifying the risks involved in work-related motions, using gesture recognition (GR) techniques to recognize professional motions and estimate their frequency and duration on the workers’ shift. Peppoloni [40] developed a monitoring system by training State machines to classify manual handling activities with data from a wearable sensor network. Likewise, Ryu [41] trained a Support Vector Machine (SVM) classifier, with data from an accelerometer placed on the wrist, to classify a mason’s actions (e.g., laying and adjusting bricks). With deep learning architectures, Slaton [42] trained a hybrid network, containing convolutional and recurrent Long Short-Term Memory (LSTM) layers, to recognize construction-related activities. Parsa [43] applied Temporal Convolutional Networks (TCNs) to segment videos and recognize manual handling tasks with different ergonomic risk levels.

Hidden Markov Models (HMMs) have been widely used for the modeling and recognition of human gestures. HMMs model the dynamic behavior of gestural time series based on a probabilistic interpretation of the gesture samples. The HMMs assume that a hidden state sequence causes the observed sequence (gesture samples). HMMs capture the motion patterns presented in the training set’s gestures, meaning that they will not recognize other variations from these patterns that could emerge during the movement performance, after the training. To address this issue, Caramiaux [44] proposed the Gesture Variation Follower (GVF), representing pre-recorded template gestures with continuous state-space models. Particle Filtering was used to update the models’ parameters to estimate the likeliest template of a new observation, considering its varying gesture characteristics. The gesture’s speed, size, scaling and rotation angles were considered the varying gesture characteristics and state variables.

Despite the fact that ergonomic evaluation based on GR adds factors such as the frequency and duration of activities into the analysis, basing the ergonomic evaluation on only these two factors could lead to the oversight of other risk factors in the motions that could cause the development of WMSDs.

## 3. Methodology

Due to the nature of the hypothesis defined for this study, a physics-based approach was selected to model the dynamics of professional movements. Physics-based approaches have proved to be capable of handling joint predictions efficiently and because of the use of a transition function, they perform well with observations obtained from different environments and subjects, without extensive training datasets. This generalization capability is essential if workers from various industrial sectors are to be monitored. Moreover, by using a physics-based model, information could be extracted regarding the human dynamics and their response to risk factors, by examining the resulting trained models.

**Figure 1:**
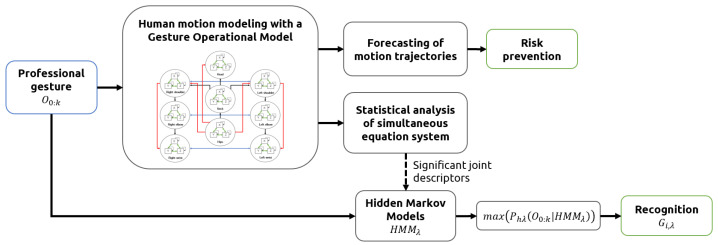
Methodology pipeline.

In this study, human motion was represented as a sequence of human poses, where each pose was described through 3D-joint angles. The modeling of each gesture was done using the Gesture Operational Model methodology [14], which was extended by integrating more assumptions into the representation of the motion of joints. The models were used to predict the trajectory of joint angles, instead of joint positions, to avoid forecasting errors such as bone stretching and invalid skeleton configurations, errors that commonly occur in models trained with joint positions [45,46,47]. The proposed methodology is illustrated in Figure 1.

The statistical significance of the assumptions of each model was computed to determine the body joints contributing the most to the professional movements. The selected joint angles were validated by comparing their gesture recognition performance with another two sensor configurations, the first using all joint angles for training, and the second using only a small set of two hand-picked sensors. Finally, the forecasting ability of the models was evaluated, and a sensitivity analysis was conducted to analyze the stability and behavior of the system when external forces affect system response, meaning a change in the posture and ergonomic risk level of the motion.

### 3.1. Data Collection and Gesture Vocabularies

#### 3.1.1. Inertial Motion Capture Technology

Due to the advantages of using motion capture (MoCap) technologies, based on inertial sensors for the MoCap of industrial workers and the subjects’ movements, the BioMed bundle motion capture system from Nansense Inc. (Baranger Studios, Los Angeles, CA, USA) was used. This system consisted of a full-body suit composed of 52 IMUs placed throughout the body and hands. The sensors allowed the orientation and acceleration of body segments on the articulated spine chain, shoulders, limbs and fingertips to be measured at a rate of 90 frames per second. Those 52 rotations were combined to create a kinematic skeleton that included the body segments measured. The Euler local joint angles on three axes X, Y, and Z were computed through the inverse kinematics solver provided by Nansense Studio (suit software). The joint angles per time frame were then exported to Biovision Hierarchy (BVH) files. Before the analysis, an offline pre-processing procedure of the data was followed. The motion data was low pass filtered to mitigate noise, and the common zero velocity update algorithm was applied to remove the drifting caused by electromagnetic interference.

#### 3.1.2. Recording and Gesture Vocabularies

**Figure 2:**
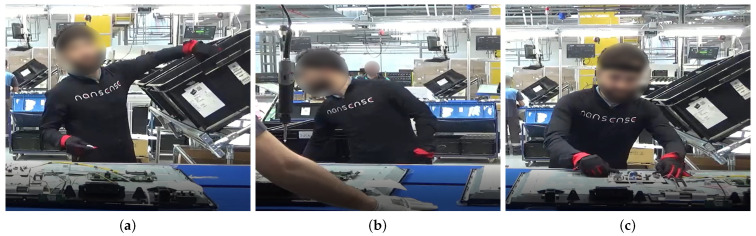
Gesture vocabulary with gestures for TV assembly (G_1_). (**a**) G_1,1_: Grab the electronic card from a container; (**b**) G_1,2_: Take a wire from a container; (**c**) G_1,3_: Connect the electronic card and wire and place them on the TV chassis.

**Figure 3:**
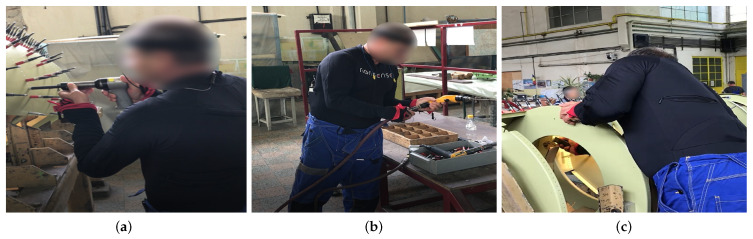
Gesture vocabulary with gestures for airplane assembly (G_2_). (**a**) G_2,1_: Rivet with the pneumatic hammer; (**b**) G_2,2_: Prepare the pneumatic hammer and grab rivets; (**c**) G_2,3_: Place the bucking bar to counteract the incoming rivet.

**Figure 4:**
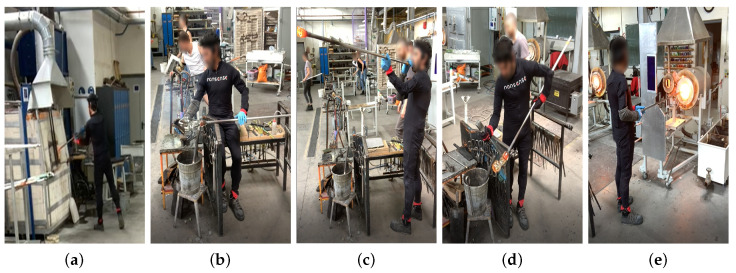
Gesture vocabulary with gestures for glassblowing (G_3_). (**a**) G_3,1_: Grab glass melt from the oven; (**b**) G_3,2_: Shape the carafe’s curves; (**c**) G_3,3_: Blow through the blowpipe; (**d**) G_3,4_: Shape the carafe’s neck with pliers; (**e**) G_3,5_: Heat the glass of the carafe.

**Figure 5:**
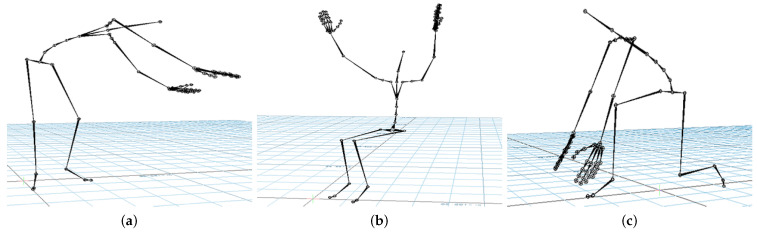
Gesture vocabulary with motion primitives based on EAWS (G_4_). (**a**) G_4,1_: Standing while bending forward and rotating the torso; (**b**) G_4,2_: Sitting while raising arms above shoulder level; (**c**) G_4,3_: Kneeling while bending forward.

Industrial workers from television (TV) production, airplane manufacturing, and glassblowing sectors were recorded under real conditions in their respective factories. Figure 2, Figure 3, Figure 4 and Figure 5 illustrate the four gestures vocabularies, and a detailed description of each gesture is provided in the Appendix A.

The professional gestures from each gesture vocabulary presented essential differences in their execution due to the different contexts in which they were recorded. For instance, G_1,1_, G_1,2_, or G_2,2_ were mostly manipulating tools or objects, where the subject grabbed an object or prepared it for later use. The iterations for these gestures had a high intra-class variance since their motion was not restricted, nor was high precision or dexterity required. On the contrary, for the gesture vocabulary G_3_ and gestures G_1,3_, G_2,1_, and G_2,3_, the subjects needed to be more precise since they placed the objects in a specific position. It has to be considered that human factors such as level of experience, fatigue, or mental stress affected how the subjects’ bodies performed the gestures. Although this did not apply for the G_3_, high dexterity and technicity were required to execute the gestures effectively. The gestures from G_3_ were recorded from a glassblowing expert, who performed the gestures with high repeatability and low spatial and temporal variations between iterations, to produce a carafe four times with the same specifications. Regarding G_4_, there was a low intra-class variation in this dataset since the performance of each movement was controlled. Feedback was provided to subjects in order to perform the gesture demanded correctly. On the other hand, the inter-class variation was intended to be low, where there were only a few variations in the postures assumed in each gesture. The end in using this last dataset was to test whether the proposed methodology was able to identify the small variations between motions and provide an accurate estimate of the joints that most contributed to the execution of the 28 motions.

From an ergonomic point of view, these four datasets could assist in the evaluation of human motions in industrial settings. The modeling of these motions could help in evaluating the subjects’ manual dexterity in relation to the gesture’s ergonomic risk. For example, as mentioned, the glassblower had a high level of dexterity for glassblowing gestures, but some observational methods could recognize that the gestures executed were ergonomically risky (e.g., G_3,3_ and G_3,4_). An ergonomic analysis of these gesture vocabularies could therefore aid in improving ergonomically how the professional gestures were executed without affecting manual dexterity.

### 3.2. Movement Representation with GOM

**Figure 6:**
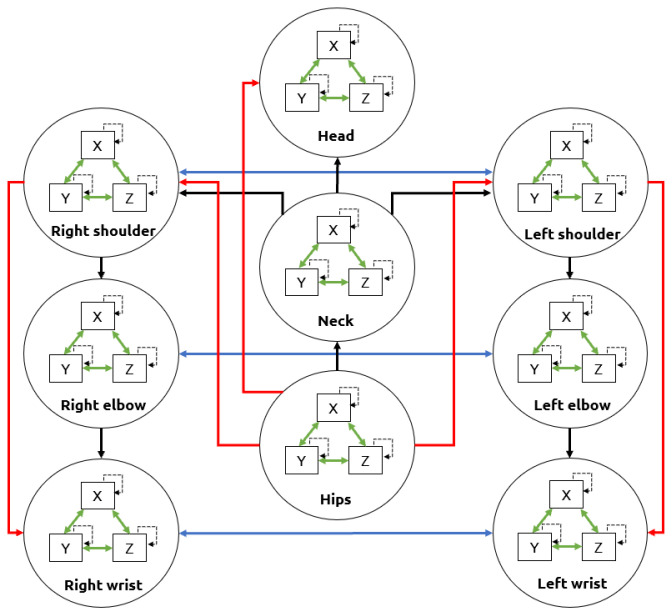
Upper-body assumptions that constitute a Gesture Operational Model. The intra-joint association is indicated by green arrows, transitioning over time with dashed arrows, inter-limb synergies with blue arrows, intra-limb serial mediation with black arrows, and intra-limb non-serial mediation with red arrows.

The Gesture Operational Model was composed of auto-regressive models that learn the dynamics of each body part. Each representation had different assumptions of the dynamic association between body parts. These assumptions consisedt of the intra-joint association (H1), inter-limb synergies (H2), serial (H3.1) and non-serial intra-limb mediations (H3.2), and transitioning over time (H4), [14]. For the intra-joint association, a bidirectional relationship was assumed between variables where the motion is decomposed e.g., joint angles on the *X*-axis, *Y*-axis, and *Z*-axis. The transitioning assumption was that current values depend on their previous values. The inter-limb synergies assumed a relationship between body parts that worked together to achieve a motion trajectory e.g., using both hands to execute a specific gesture. Finally, the serial and non-serial intra-limb mediations included the relationship between joints, whether directly and not directly connected e.g., the wrist was directly connected with the elbow (serial mediation) and indirectly connected with the shoulder (non-serial mediation). These assumptions are represented in Figure 6.

**Figure 7:**
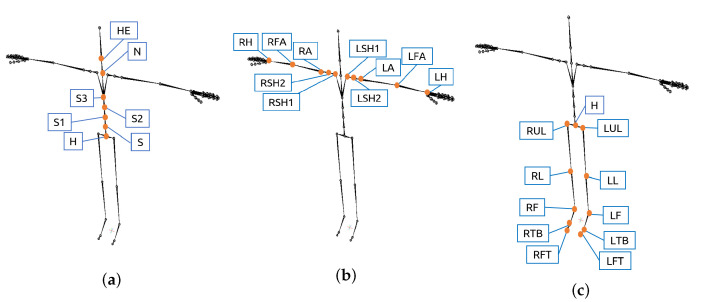
Location of the sensors that provide the XYZ joint angles included in GOM’s state-space equations. (**a**) Spine parts; (**b**) Arm parts; (**c**) Leg parts.

The number of representations was equal to the number of associated dimensions for a given body part, multiplied by the number of body parts defined in the GOM. The representation of each body part had different assumptions depending on its location within the body:

- Intra-joint association: All body parts included it in their representation.
- Inter-limb synergies: Only the body parts representing joint angles from arm and leg parts included this assumption.
- Intra-limb serial and non-serial mediation: The assumptions included in each representation depended on the body part location within the body. The joint angles related to the spine only included in their equation joint angles of other spine parts with which it had serial or non-serial mediation. The serial and non-serial mediations from angles related to the spine are illustrated in Figure 7a. The angles related to the arms only included in their equation joint angles of other arm parts with which it had serial or non-serial mediation (Figure 7b). Equally, the angles related to the legs only included in their equation joint angles of other leg parts (Figure 7c).

The transitioning assumptions corresponded to the lagged endogenous variables, where lag depended on the order given to the model. For this work, second-order autoregressive models were selected. The order was selected according to the correlation between lag values in the time series (auto-correlation). If the observations had positive auto-correlations with a certain number of lags, then it was better to have a higher order of differencing until the auto-correlation was negative and more than −0.5, to avoid overdifferencing [48].

An example of a mathematical representation of the assumptions is shown in Equation (2), for a motion on the *X*-axis ($X_{ax}$) of a body part *P*, with only two dimensions $X_{ax}$ and $Y_{ax}$, and assumptions that includes an association with only a second body part ($P_{2,X_{ax}}\left(t-1\right)$).

(2) $$P_{1,X_{ax}}\left(t\right)=\underset{H1}{\underbrace{P_{1,Y_{ax}}\left(t-1\right)}}+\underset{H2}{\underbrace{P_{2,X_{ax}}\left(t-1\right)}}+\underset{H4}{\underbrace{P_{1,X_{ax}}\left(t-1\right)}}+\underset{H4}{\underbrace{P_{1,X_{ax}}\left(t-2\right)}}$$

These representations were then translated into simultaneous equations by using state-space modeling. State-space equations allowed estimation of the state of the system according to the input-output data [49]. Thus, given the input and the current state of the system, state-space gave the hidden states that resulted in the observable variables. A state-space representation is shown in Equations (3) and (4). Equation (3) is the state-space equation, a first-order Markov process where *A* is the transition matrix. Equation (4) is the measurement equation, where the time derivative of the state vector s(t) is taken into account for the computation of the output y(t) along with the input vector u(t), where *C* is the output matrix and *D* the feed-through matrix.

(3) $$s\left(t\right)=AS_{s}\left(t-1\right)+w\left(t\right)$$

(4) y(t)=Cs(t)+Du(t)

To model the GOM representation of the Equation (2) using second-order state-space modeling, first, the state-space variable is substituted with the subtraction of two previous values of the body part to model, each multiplied by one coefficient of the transition matrix:

(5) $$s\left(t\right)=AS_{s}\left(t-1\right)=\left[\begin{array}{cc} \alpha_{1} & 0\\ 0 & \alpha_{2} \end{array}\right]\left[\begin{array}{c} P_{1,X_{ax}}\left(t-1\right)\\ -P_{1,X_{ax}}\left(t-2\right) \end{array}\right]=\left[\begin{array}{c} \alpha_{1}P_{1,X_{ax}}\left(t-1\right)\\ -\alpha_{2}P_{1,X_{ax}}\left(t-2\right) \end{array}\right]$$

For the measurement equation, the input vector u(t) corresponds to the endogenous variables, for the case of Equation (2), it consists of the intra-joint association and inter-limb synergy:

(6) $$P_{1,X_{ax}}\left(t\right)=\left[\begin{array}{cc} 1 & 1 \end{array}\right]s\left(t\right)+\alpha_{3}P_{1,Y_{ax}}\left(t-1\right)+\alpha_{4}P_{2,X_{ax}}\left(t-1\right)$$

Finally, by merging Equations (5) and (6), the state-space representation is obtained:

(7) $$\begin{array}{cc} P_{1,X_{ax}}\left(t\right)= & \alpha_{1}P_{1,X_{ax}}\left(t-1\right)-\alpha_{2}P_{1,X_{ax}}\left(t-2\right)+\\ & \alpha_{3}P_{1,Y_{ax}}\left(t-1\right)+\alpha_{4}P_{2,X_{ax}}\left(t-1\right) \end{array}$$

The full body modeling consisted of three sets of equations for each body part, one for each dimension X, Y, and Z. Hence, by discarding the body parts from the fingers, the GOM consisted of 84 equations per gesture. The coefficients of the equation system were estimated using the Maximum Likelihood Estimation (MLE) via Kalman filtering [50]. For the coefficient estimation, first, the probability of obtaining the observation vectors $O_{0:k}$ was defined:

(8) $$P\left(O_{0:k}\right)=\prod_{t=0}^{k}P(O_{t}|O_{0:t-1})$$

which consisted of the products of probabilities of the observation at time *t*, given previous observations. This probability distribution is considered Gaussian, as shown in the following equation:

(9) $$P\left(O_{0:k}|\psi \right)=\prod_{t=1}^{k}\exp\left\{-\frac{{\left(o_{t}-{\tilde{o}}_{t}^{t-1}\right)}^{2}}{2F_{t}^{t-1}}\right\}{\left(2\pi \left|F_{t}^{t-1}\right|\right)}^{-\frac{1}{2}}d^{\prime }$$

where $F_{t}^{t-1}$ is the covariance and ${\tilde{o}}_{t}^{t-1}$ is the mean. From Equation (9), the log-likelihood was computed, where the Kalman filter could optimally estimate the mean and covariance that gave the maximum likelihood:

(10) $$\log L\left(\psi |O_{0:t-1}\right)=-\frac{k}{2}\log2\pi -\frac{1}{2}\sum_{t=1}^{k}\log\left|F_{t}^{t-1}\right|-\frac{1}{2}\sum_{t=1}^{k}\frac{{\left(o_{t}-{\tilde{o}}_{t}^{t-1}\right)}^{2}}{F_{t}^{t-1}}$$

The Kalman filtering consisted of two steps which were repeated until obtaining the maximum likelihood. These are known as the prediction and update steps. Initial values were set, then the log-likelihood was computed for the evaluation in the prediction step. Next, in the update step, the variance and mean were updated according to the Kalman gain ($K_{t}$), until, in the prediction step, the maximum likelihood was achieved:

(11) $$K_{t}=\frac{F_{t}^{t-1}}{\left(F_{t}^{t-1}+R\right)}$$

(12) $$\begin{array}{cc} {\tilde{o}}_{t}^{t-1}={\tilde{o}}_{t}^{t-1}+K_{t}\left(o_{t}-{\tilde{o}}_{t}^{t-1}\right) & \;\;F_{t}^{t-1}=F_{t}^{t-1}-K_{t}F_{t}^{t-1} \end{array}$$

In the end, the computation of the coefficients of the state space models was derived through Equation (10).

### 3.3. Applications of the GOM

#### 3.3.1. Selection of Significant Joint Angles

Statistical analysis was done to investigate the significance of the model assumptions in relation to the body part associations defined within the GOM. By estimating the statistical significance of each assumption, it was possible to determine which joint descriptors contributed the most to the execution of all the gestures of each gesture vocabulary. The number of times a joint descriptor was statistically significant in all the equations that constitute the GOM was counted in order to select the most important joint angles for each gesture vocabulary.

In order to evaluate the selection of the most meaningful joint angles, different combinations from the selected joint angles were used to train Hidden Markov Models (HMM) for gesture recognition using an “all-shots” approach. The motion data of sensors that provided at least one of the top three joint angles contributing the most in the response for the spine, arms, and legs parts motion was used for gesture recognition. Since one sensor provided three angles of one joint, all the joint angles of the sensor were used for training. The first combination to test for gesture recognition consisted of a minimal sensor configuration: the best sensor to measure the spine, another which was the best to measure the arms, and a third for the legs. If the recognition performance was low, an extra sensor was added to the configuration to improve the performance, or it was replaced by another of the top three sensors selected to measure its corresponding body location (spine, arms, or legs). The configuration that achieved the best performance was compared with the recognition performance obtained using all the joint angles of the sensors. The recognition performance, by using only a minimal set of two sensors, was also computed for comparison. This minimal set consisted of two hand-picked sensors, which provided the Euler joint angles of the right forearm (RFA) and hips (H). The sensor placed on the right forearm was chosen since most of the subjects in all datasets were right-handed, and the hips sensor was chosen because the origin of all movement of the spine starts from the hips.

To determine the best HMM setting for each gesture vocabulary, both ergodic and left-right topologies were tested, in addition to a different number of hidden states. The performance metric used consisted of the F-score. In the training phase of HMM, each professional gesture G_*v*,*c*_, where $v\in \left[1,4\right]$ indicates the gesture vocabulary and c∈N the gesture of the G_*v*_, is associated to an HMM. The set of models for all gestures for every gesture vocabulary is $G_{v\in \left[1,4\right]}={\left\{HMM_{c}\right\}}_{c\in N}$.

#### 3.3.2. Prediction of Joint Angles Trajectories

For evaluating the forecasting performance of the GOM models, the joint angle sequences of each gesture were simulated by solving the simultaneous equation system of the GOM. The models forecasted one time frame per iteration, then, after forecasting all the time frames of the gesture, the simulated gesture was compared with the original for evaluation. Consequently, their forecasting ability was evaluated by computing Theil’s inequality coefficient (U) along with its decompositions: bias proportion (U_B_), variance proportion (U_V_), and covariance proportion (U_C_).

A sensitivity analysis was conducted to investigate the reaction of the models after a shock occurred in one of their variables. For this analysis, a disturbance of 80% was applied only in the first two frames of the gesture, then the whole gesture was forecasted. This analysis aimed to simulate the situation where subjects were exposed to external forces that affected their performance or made the workers assume awkward postures that increased the risk of injury.

## 4. Experimental Results

### 4.1. Statistical Significance of Motion Descriptors

Here, an example of a joint angle motion equation for one gesture from each vocabulary will be provided. These examples are offered to enable visualization of the coefficients and *p*-values of the different assumptions that compose the equation, where some variables need to remain dynamic and others static. The first example is for the equation of the gesture G_1,1_ (grab an electronic card from a container) for the joint angle $RA_{Y}$, which is the joint angle of the right arm on the Y-axis:

(13) $$\begin{array}{cc} RA_{Y}\left(t\right)= & \underset{p=0.01}{\underbrace{\left(-86.76\right)LSH1_{Y}\left(t-1\right)}}+\underset{p=0.001}{\underbrace{\left(-169.03\right)LSH1_{Z}\left(t-1\right)}}+\\ & \underset{p=0.008}{\underbrace{\left(88.48\right)LSH2_{X}\left(t-1\right)}}+\underset{p=0.001}{\underbrace{\left(-67.38\right)RSH1_{Y}\left(t-1\right)}}+\\ & \underset{p=0.002}{\underbrace{\left(-142.13\right)RSH1_{Z}\left(t-1\right)}}+\cdots +\underset{p=0.508}{\underbrace{\left(-2.18\right)RA_{X}\left(t-1\right)}} \end{array}$$

By doing a statistical analysis of Equation (13), the *p*-values show intra-limb serial mediations with the joint angles on the *Y* and *Z*-axis of the left shoulder (LSH1) and intra-limb non-serial mediation with the right shoulder (RSH1). In the last equation, it should be noted that there is no intra-joint association shown by the *p*-value of the $RA_{X}$, and although it is not illustrated in the equation, there is no inter-limb synergy either. These results make sense since most of this motion is highly dependent on movements of the shoulders. Consequently, it is the reason that shoulders are statistically significant for the equation of $RA_{Y}$. The second example is the equation for G_2,3_ (Hold the bucking bar) for the joint angle of the neck on the *X*-axis ($N_{X}$):

(14) $$\begin{array}{c} N_{X}\left(t\right)=\underset{p=0.001}{\underbrace{\left(-1.2\right)N_{Y}\left(t-1\right)}}+\underset{p=0.001}{\underbrace{\left(-0.47\right)N_{Z}\left(t-1\right)}}+\\ \underset{p=0.002}{\underbrace{\left(-0.01\right)S2_{X}\left(t-1\right)}}+\underset{p=0.001}{\underbrace{\left(-0.02\right)S2_{Y}\left(t-1\right)}}+\\ \underset{p=0.001}{\underbrace{\left(-0.01\right)S3_{X}\left(t-1\right)}}+\cdots +\underset{p=0.84}{\underbrace{\left(0.01\right)H_{X}\left(t-1\right)}} \end{array}$$

Equation (14) indicates that there is an intra-joint association with $N_{Y}$ and $N_{Z}$, and an intra-limb serial mediation with the S3. There is an intra-limb non-serial mediation with S2, but not with H. For the gesture of holding a bucking bar to counteract a rivet, it is necessary to bend forward on the *X*-axis and *Y*-axis, which corresponds to what Equation (14) shows, that is to say, that joint angles from S2 and S3 on the *X* and *Y*-axis are statistically significant and contribute to gesture. Moreover, for this gesture, the subject needed to rotate the neck to see where to place the bucking bar; therefore, this matches with the intra-joint association indicated by the *p*-value of $N_{Y}$ and $N_{Z}$.

The next equation is an example of gesture G_3,2_ (shape the carafe curves) for the joint angle of the left shoulder on the *X*-axis, representing the motion of the left clavicle ($LSH2_{X}$):

(15) $$\begin{array}{c} LSH2_{X}\left(t\right)=\underset{p=0.003}{\underbrace{\left(0.15\right)LSH2_{Y}\left(t-1\right)}}+\underset{p=0.016}{\underbrace{\left(0.17\right)LSH2_{Z}\left(t-1\right)}}+\\ \underset{p=0.001}{\underbrace{\left(-0.02\right)LA_{Y}\left(t-1\right)}}+\underset{p=0.001}{\underbrace{\left(-0.36\right)RSH2_{X}\left(t-1\right)}}+\\ \underset{p=0.001}{\underbrace{\left(-1.05\right)RSH2_{Z}\left(t-1\right)}}+\cdots +\underset{p=0.731}{\underbrace{\left(-0.01\right)LFA_{X}\left(t-1\right)}} \end{array}$$

Statistical analysis of the Equation (15) indicates an intra-joint association, intra-limb serial mediation with the left arm, and an inter-limb synergy with the right shoulder. In this gesture, both arms must cooperate to shape the carafe correctly. The joints angles from the right shoulder contribute to the response of the left shoulder, since with the right arm the glassblower shaped the curves of the carafe, while the left arm slowly rolled the blowpipe. The Equation (16) presents a gesture from the G_4_, where the subject bent forward more than $60^{\circ }$ for the joint angle S3 on the *Y*-axis ($S3_{Y}$):

(16) $$\begin{array}{c} S3_{Y}\left(t\right)=\underset{p=0.007}{\underbrace{\left(2.13\right)S3_{X}\left(t-1\right)}}+\underset{p=0.001}{\underbrace{\left(-0.17\right)S3_{Z}\left(t-1\right)}}+\\ \underset{p=0.012}{\underbrace{\left(-0.91\right)H_{X}\left(t-1\right)}}+\underset{p=0.001}{\underbrace{\left(0.42\right)S1_{Y}\left(t-1\right)}}+\\ \underset{p=0.001}{\underbrace{\left(-3.24\right)S2_{X}\left(t-1\right)}}+\cdots +\underset{p=0.061}{\underbrace{\left(-0.06\right)HE_{X}\left(t-1\right)}} \end{array}$$

The *p*-values show that there is a dependency on the intra-joint association assumption. The joint angles on the *X*-axis from the sensors S3, *H*, and S2 are statistically significant and have the highest coefficient values, which is to be expected since the spine moves on the *X*-axis in order to bend forward. Moreover, there is an intra-limb serial and non-serial mediation with joint angles on the *Y*-axis, except for $H_{Y}$.

The top ten variables that contributed the most in the gestures of each gesture vocabulary are illustrated in Table 1, Table 2, Table 3 and Table 4. From these joint angles, as mentioned in the methodology, different sets are used for gesture recognition. The results are shown in Section 4.2.

### 4.2. Validation of the Joint’s Selection

In this section, the recognition performances achieved by the different sets of sensors are reviewed and compared. Table 5 summarizes the results obtained when using different sensors and shows the configuration selected according to the GOM (SS), which achieved the best recognition performance. The gesture vocabulary G_1_, as mentioned earlier, was composed of three gestures, each with 106 repetitions. HMMs with seven hidden states achieved the best recognition performance, trained with the joint angles provided by the sensors S1, LA, and RUL, selected by using the GOM. The precision and recall achieved with each configuration of sensors to recognize gestures from G_1_ are illustrated in Table 6 and Table 7.

G_2_ contained three gestures with 10 to 12 repetitions each. HMM with eight hidden states achieved the best performance. The SS sensor configuration had the best F-score, as shown in Table 5, 5.44% more than the set with the two sensors, and 22.31% more than the set with all the sensors. Table 8 and Table 9 shows the recognition performance for G_2_ with each sensor configuration, where the best precision and recall was achieved by the set SS.

G_3_ consisted of five gestures with 10 to 35 repetitions for each. For this gesture vocabulary, HMM with four states modeled the best gestures and yielded the best recognition performance. This performance is illustrated in Table 10 and Table 11. The configuration of sensors selected using GOM improved the overall F-score by at least 6% over the other sets. The G_4_ was composed of the 28 motion primitives based on EAWS, where each exposed the subjects to different ergonomics risks concerning the posture. There are 30 repetitions for each motion, and HMM with seven states modeled the best gestures from G_4_. For the gesture recognition of the 28 classes, the set SS yielded the higher F-score (91.77%), average precision (91.90%) and recall (92.33%), over the minimized set of two sensors and the set with all sensors. The minimized set achieved an average precision of 74.16% and an average recall of 77.31%. By using all the sensors for the recognition, an average precision of 84.76% and an average recall of 86.46% was achieved.

#### Performance Analysis of Selected Sensors Sets

The relevance of the sensors selected for G_1_ in the execution of the three gestures was proven due to the high recognition performance achieved. By observing the results for G_1_ in Table 6 and Table 7, it became apparent that the three sensors chosen improved the precision of the recognition and the recall of the gestures G_1,2_ and G_1,3_. In the case of G_1,1_, the two sensors configuration had the best recall. Overall, the selected sensors had the best performance, with at least +1.2%. From the four gesture vocabularies, G_1_ had the best performance for gesture recognition, which could be due to the low inter-class variance between the three gestures.

G_2_ was the gesture vocabulary with the highest number of sensors selected. The reason could be because the gestures in this vocabulary were more complex and more prolonged. The most complicated gesture to model and recognize was G_2,2_, which was expected since it is the gesture that could vary the most in its execution (high intraclass variance) from among the three gestures. The subject did not prepare the material in exactly the same way for each iteration. The subject was slower in some iterations than others since he required more time to adjust the pneumatic hammer or needed to prepare more rivets. The low intra-class variance could be because of the way the gestures were executed, which depended on the locations where the worker was going to fasten the metal plate with the rivets. For the recording of G_2_, there was only one airplane structure to build, and there were no iterations where the subject placed the pneumatic hammer in the same location more than once.

The sensors selected for recognition of gestures from G_3_ were validated by achieving a high recognition performance of the five gestures. By analyzing the results in Table 10 and Table 11, the recall is improved in most gestures using the set SS, since the selected sensors capture the motion better. Regarding precision, the set SS improved it for G_3,4_ and G_3,5_, but then it decreased in comparison with the minimized set for G_3,1_. This could be because the information provided by the sensors S3 and LSH2 generated similar patterns between the gestures G_3,1_ and G_3,3_. The minimized set had the worse precision and recall for G_3,4_. The reason could be because of the lack of information on the motion of the shoulders. According to GOM, the shoulders contribute most to executing this gesture. Four out of the five gestures in this vocabulary generated similar patterns on the shoulder and arms. Still, there was low intra-class variation because of the high level of the subject’s dexterity, as an expert in glassblowing. In addition, the subject used a metal structure for shaping the carafe that limited any potential freedom in the gesture performances. Finally, for G_4_, a maximum F-score of 91.77% was recorded for the recognition of 28 motion primitives, using the selected sensors S2, LA, RS1, RUL and LFA. The poor performance of the minimized set was due to its failure to discriminate between motions that vary only with regard to posture of the legs, while the poor performance using all the sensors can be explained by the multiple dimensions of the data.

### 4.3. Simulated Movements and Sensitivity Analysis

**Figure 8:**
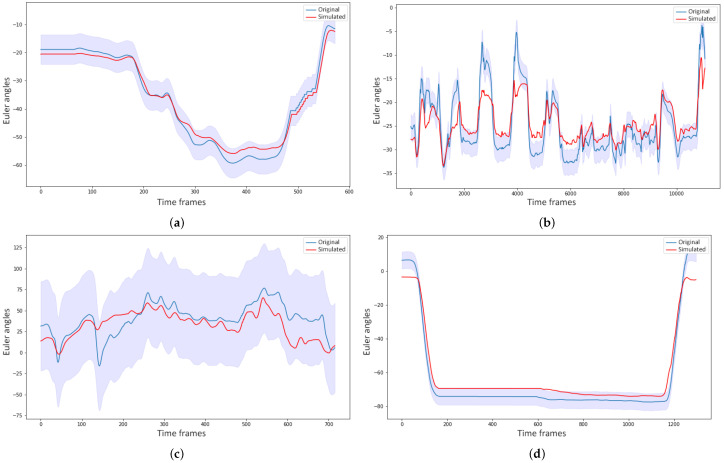
Examples of simulated gestures, their original gesture, and confidence bounds of 95% (**a**) Simulation of the gesture G_1,3_ on the joint angle LA_X_; (**b**) The simulated joint angle sequence of S_Z_ for G_2,3_; (**c**) Simulation of LFA_Y_ for the gesture G_3,1_; (**d**) Forecasting of RA_X_ for G_4,9_, which consists of raising the forearms above the shoulder level.

This section presents the results of the trajectory prediction and sensitivity analysis. Figure 8 illustrates one example of a simulated gesture and the original from each gesture vocabulary, with confidence bounds of 95%. Figure 8b,c show that the models could capture the patterns generated on the motion of the spine by the task of buckling a rivet and the motion of the forearm while the glassblower was rotating the blowpipe. For more quantitative measurement of the forecasting performance, the Theil inequality coefficient, its decompositions, and the root mean square error were computed. Table 12 shows the forecasting performance for one gesture of each vocabulary on three Euler angles of a joint used during the execution of the gesture. By observing the Figure 8 and Table 12 alone, it can be assumed that the forecasting performance was good for these gestures. The original and simulated values were close to each other, and the simulated values were mostly inside the confidence bounds.

**Figure 9:**
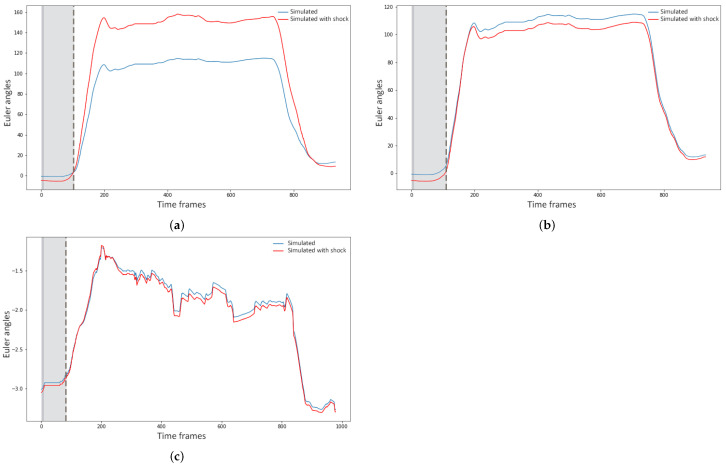
Simulated joint angles with and without disturbance of 80% on the two initial time frames. (**a**) Simulation of the joint angle LA_X_ with a disturbance on the joint angles of LSH2 (blue) and without (red); (**b**) Simulated joint angle sequence of LA_X_ with a disturbance on the joint angles of RSH2 (blue) and without (red); (**c**) Simulation of the joint angle S2_Y_ with a disturbance on the joint angles of H (blue) and without (red).

In Figure three examples of shocks applied to different variables for the sensitivity analysis are illustrated. Figure 9a,b show the forecasting behavior of the model of the joint angle LA_X_ for the gesture of raising the hands above the shoulder level. In Figure 9a a shock was applied on the joint angles of LSH2, and in Figure 9b, it was on the joint angles of RSH2. It is apparent that applying a shock to the left shoulder affected the motion of the left arm far more than applying it to the right shoulder, due to the strong mediation of the left shoulder over the left arm motion. Figure 9c shows the simulated motion of S2_Y_ when the subjects rotated their torso to the left. The shock in this case was applied to the joint angles of H. It can be seen from the figure that the model was able to adapt fast and, indeed, in less than 1 s (90 frames), which indicated low sensitivity of the model to external disturbance. However, there was still a small variation in the forecasting if compared to the simulated gesture forecast without shocks.

## 5. Discussion

This paper evaluates GOM’s feasibility to model industrial workers and subjects’ dynamics and select the joint angles that best represent the gesture vocabulary, and predict their joint angles’ trajectory. The statistical analysis conducted on the GOM models permitted identification of the joint angles that contributed most to the execution of the gestures of each vocabulary. For validation, these joint angles were then used for gesture recognition. These results demonstrate the potential of the selected set of sensors for a posture-based ergonomic analysis. By only using the data of the selected sensors, it was possible to discriminate accurately between different professional gestures and motion primitives where various postures of the spine, arms, and legs were assumed. The recognition of these changes in posture are clearly useful for ergonomic analysis of professional gestures. By applying a whole gesture to the trained HMMs from this vocabulary, the models could tell whether an awkward motion primitive is performed and which body part causes this ergonomic risk (i.e., spine, legs, or arms).

By solving the simultaneous equations that compose the GOM, it was possible to accurately forecast the modeled gesture, using Euler joint angles as input. Moreover, the models are tolerant to small variations in the gestures and offsets between same class gestures, which could be due to different recording conditions (different subjects or different recording days). Regarding the sensitivity analysis, the models showed low sensitivity to external disturbances, with only a small variation in the forecasting from that of a simulation without shocks. The response of the models to the shocks applied on different variables could be useful for detecting any physical strain (e.g., on the shoulders or lower back) or a load that affects the workers’ performance and increases the ergonomic risk of the motion.

The industry has used ergonomic evaluations based on joint angle thresholds widely, due to their practicality. Previous studies have applied these evaluations in their analysis, where their only contribution was to fill them automatically using motion capture technologies [10,11,12,13]. An ergonomic analysis using such an approach can result in over-simplicity and ignore other potential risks workers are exposed to (e.g., external forces and dangerous movements). Menytchas [17] expanded such ergonomic analysis by examining the kinematics and kinetics of professional movements to identify joints that accumulate the most strain. The kinetic descriptors used in that study, however, did not allow for accurate discrimination between dangerous motions with small variations in the posture; moreover, they do not analyze the dynamics of movements, unlike the present study, which allowed for a good recognition performance and distinction between motions of different ergonomic risk.

In this study, GOM was proved to be useful for ergonomic analysis of professional motions. In comparison with the approach taken in the previous study by Manitsaris [14], a more in-depth analysis was conducted over the dynamic relationships of body parts, including more assumptions in the mathematical representation of each body joint motion. This gave insight into the influence of all body parts that work together to execute a specific movement and into devising helpful strategies to address ergonomic hazards, such as optimizing workspaces. Moreover, the methodology which was followed allowed the selection of the most meaningful joint angles for gesture recognition, improving the recognition performance considerably.

Despite the good performances and contributions achieved, this study highlighted some limitations regarding the use of inertial sensors in real workplace scenarios. Inertial sensors can offer precise and reliable measurements to study human motion; however, the degree of this precision and reliability depends on the site, movements, and tools handled during the performance. For instance, in the recording for the gesture vocabularies G_2_ and G_3_, workers used plastic gloves or did not wear the gloves that come with the inertial suit in order to avoid disturbances in the measurements. For this study, the motion data needed to be pre-processed after the recording to remove disturbances and drifts that could affect the results of the method.

## 6. Conclusions

From the literature reviewed, most of the studies used inertial sensors for quantifying the intensity, repetition, and duration of extreme motions and postures. The ability to extract information about work content from kinematics data is underutilized. Industrial workers perform complex professional gestures that contain crucial information about ergonomic risks. In this paper, not only was the contribution of every body joint in the execution of a specific professional gesture statistically estimated, but how they all operationally cooperate was modeled using GOM, and, in addition, their motion trajectories were accurately predicted using the trained models. GOM is based on state-space representations and consists of a simultaneous equation system of differential equations for all body body parts. The most significant joint motions for each gesture vocabulary were selected based on their statistical significance in the GOM models. The selection was then validated by achieving a high recognition performance of gestural time-series, which was modeled using continuous HMM. Four datasets were created for this work that contain professional gestures recorded under real conditions in factories and in a laboratory environment. The forecasting performance of the models was evaluated by comparing the simulated gestures with their original values. According to the Theil inequality coefficient and its decompositions, the performance of the models can be considered accurate.

Analyzing the response of the models to external disturbances and identifying the body joints to enable tracking for ergonomic monitoring could be useful for faster and more efficient evaluation of workers’ gestures. Furthermore, the models could be used for ergonomic risk prevention. They could detect patterns in the motion trajectories that imply exposure to an ergonomic risk factor (e.g., workers are bending their torso or raising their arms beyond a level that could be considered ergonomically safe).

Lastly, using a full-body mocap suit in an industrial context has several difficulties. This study contributes to the literature by identifying the minimum motion descriptors to measure. This allows for the use of less intrusive technologies, such as smartphones and smartwatches, to measure these same motion descriptors. Future work will consist of adding kinetic measures to the assumptions that GOM models are composed of (e.g., joint moments), to complement the kinematic information, and will consider the effect of loads on the kinetic variables, which could indicate worker exposure to higher ergonomic risk.

## Figures and Tables

**Table 1:** G_1_: Televison assembly.

*p*-Value < 0.05					
Spine		Arms		Legs	
Variable	Count	Variable	Count	Variable	Count
S1_Z_	49	LA_X_	56	RUL_Y_	32
S2_Z_	47	RSH1_X_	55	LUL_Z_	32
H_Y_	46	RSH2_Y_	55	LUL_Y_	31
H_Z_	45	RSH1_Z_	54	RL_Y_	31
N_Y_	44	RSH2_Z_	53	LUL_X_	29
S1_X_	43	RSH2_X_	53	LL_X_	29
H_Z_	42	RA_Y_	49	RL_X_	29
N_X_	42	LFA_Z_	48	H_X_	29
S1_Y_	41	LSH1_X_	46	RUL_X_	29
S3_X_	41	LFA_X_	42	RUL_Z_	29

**Table 2:** G_2_: Airplane assembly.

*p*-Value < 0.05					
Spine		Arms		Legs	
Variable	Count	Variable	Count	Variable	Count
S3_X_	209	LSH2_X_	243	LUL_Z_	39
S3_Y_	205	LSH1_X_	236	RUL_X_	39
S2_X_	202	RA_Z_	230	H_X_	38
H_Z_	202	LFA_X_	229	LL_X_	38
H_X_	201	RFA_Y_	227	LUL_Y_	38
S_X_	201	LA_Y_	224	LL_Y_	37
S1_Y_	197	LA_Z_	217	LL_Z_	37
S1_Z_	193	RSH1_X_	217	RL_X_	36
S3_Z_	193	LFA_Y_	216	RL_Y_	36
N_Y_	193	LFA_Z_	212	RL_Z_	36

**Table 3:** G_3_: Glassblowing.

*p*-Value < 0.05					
Spine		Arms		Legs	
Variable	Count	Variable	Count	Variable	Count
S3_X_	155	LSH2_Y_	99	H_Y_	65
S3_Y_	155	RA_X_	92	LL_Z_	63
S3_Z_	149	RFA_Z_	90	LL_Y_	62
S2_X_	118	LSH2_X_	89	RL_X_	60
S2_Z_	116	RSH1_Z_	88	RL_Y_	60
S2_Y_	110	LSH1_Z_	86	H_Z_	59
S1_Y_	105	RSH1_Y_	85	LL_X_	59
S1_X_	102	RSH2_X_	85	RUL_Y_	58
S1_Z_	93	LA_Y_	84	RUL_Z_	58
N_X_	89	LSH2_Z_	84	LUL_Y_	57

**Table 4:** G_4_: Motion primitives based on EAWS.

*p*-Value < 0.05					
Spine		Arms		Legs	
Variable	Count	Variable	Count	Variable	Count
S3_Z_	332	LSH1_X_	534	RUL_Z_	474
S2_Y_	330	LA_X_	533	RUL_Y_	473
S2_Z_	330	RSH1_X_	523	LUL_Y_	472
S3_X_	326	LSH1_Y_	520	RL_X_	468
S3_Y_	316	LFA_X_	520	LL_X_	465
S2_X_	311	RSH2_X_	518	LUL_X_	461
HE_Z_	279	RSH1_Y_	516	LUL_Z_	457
S_Z_	264	RA_X_	514	RUL_X_	456
H_Y_	261	RSH1_Z_	508	LFT_Z_	455
N_Z_	258	LA_Y_	507	RFT_Y_	455

**Table 5:** Recognition performance with each configuration of sensors. MS: Minimal set of two sensors. SS: Selected sensors by using the GOM.

Gesture Vocabularies	$N^{\circ }$ Classes	Sensors	F-Score (%)
G_1_: TV assembly	3	MS: H and RFA	95.59
		SS: S1, LA, RUL	96.84
		All sensors	93.39
G_2_: Airplane assembly	3	MS: H and RFA	88.89
		SS: S3, S2, LSH1, LSH2, RA, LUL, RUL	94.33
		All sensors	72.02
G_3_: Glassblowing	5	MS: H and RFA	88.03
		SS: S3, LSH2, H, RFA	94.70
		All sensors	80.68
G_4_: Motions based on EAWS	28	MS: H and RFA	73.85
		SS: S2, LA, RSH1, RUL, LFA	91.77
		All sensors	84.88

**Table 6:** Recall achieved for G_1_ using HMMs.

Sensors	Recall (%)		
	G_1,1_	G_1,2_	G_1,3_
MS: H and RFA	97.17	95.28	94.34
SS: S1, LA, RUL	94.34	99.06	97.17
All sensors	92.45	95.28	92.45

**Table 7:** Precision achieved for G_1_ using HMMs.

Sensors	Precision (%)		
	G_1,1_	G_1,2_	G_1,3_
MS: H and RFA	95.37	96.19	95.24
SS: S1, LA, RUL	98.04	95.45	97.17
All sensors	94.23	91.82	94.23

**Table 8:** Recall achieved for G_2_ using HMMs.

Sensors	Recall (%)		
	G_2,1_	G_2,2_	G_2,3_
MS: H and RFA	83.33	83.33	100.00
SS: S3, S2, LSH1, LSH2, RA, LUL, RUL	100.00	83.33	100.00
All sensors	66.67	50.00	100.00

**Table 9:** Precision achieved for G_2_ using HMMs.

Sensors	Precision (%)		
	G_2,1_	G_2,2_	G_2,3_
MS: H and RFA	83.33	83.33	100.00
SS: S3, S2, LSH1, LSH2, RA, LUL, RUL	85.71	100.00	100.00
All sensors	57.14	60.00	100.00

**Table 10:** Recall achieved for G_3_ using HMMs.

Sensors	Recall (%)				
	G_3,1_	G_3,2_	G_3,3_	G_3,4_	G_3,5_
MS: H and RFA	100.00	100.00	72.72	70.00	94.29
SS: S3, LSH2, H, RFA	83.33	95.45	100.00	100.00	97.14
All sensors	70.00	100.00	45.45	80.00	97.14

**Table 11:** Precision achieved for G_3_ using HMMs.

Sensors	Precision (%)				
	G_3,1_	G_3,2_	G_3,3_	G_3,4_	G_3,5_
MS: H and RFA	90.90	95.65	100.00	70.00	91.67
SS: S3, LSH2, H, RFA	100.00	95.45	81.82	100.00	97.14
All sensors	77.78	95.65	100.00	80.00	82.93

**Table 12:** Forecasting performance of one gesture for each gesture vocabulary.

Gestures	Joint Angles	Theil Inequality U	Bias Proportion U_B_	Variance Proportion U_V_	Covariance Proportion U_C_	RMSE
G_1,3_	LSH1_X_	0.0174	0.2499	0.0030	0.7483	0.0958
	LSH1_Y_	0.0069	0.0000	0.0021	0.9996	0.0078
	LSH1_Z_	0.0147	0.0006	0.0001	1.0010	0.0083
G_2,1_	RSH2_X_	0.0939	0.0002	0.0769	0.9230	0.0648
	RSH2_Y_	0.0142	0.0000	0.0000	1.0001	0.0075
	RSH2_Z_	0.0247	0.0002	0.0018	0.9981	0.0093
G_3,4_	LSH2_X_	0.2061	0.2786	0.0275	0.6947	0.2139
	LSH2_Y_	0.3958	0.2327	0.0038	0.7645	0.1821
	LSH2_Z_	0.3662	0.4919	0.1726	0.3361	0.6323
G_4,3_	S2_X_	0.0077	0.0290	0.0187	0.9531	0.0742
	S2_Y_	0.0351	0.0906	0.2100	0.7001	0.1434
	S2_Z_	0.0115	0.0692	0.0599	0.8717	0.0776

## Data Availability

All recorded data are available upon request.

## Abbreviations

The following abbreviations are used in this manuscript:

WMSD	Work-related musculoskeletal disorders
GOM	Gesture Operational Model
IMUs	Inertial Measurement Units
HMMs	Hidden Markov Models
MS	Minimum set of two sensors
SS	Selected sensors by using a GOM

## Appendix A. Description of the Datasets’ Gestures

This appendix presents a detailed description of the gestures from the four gesture vocabularies recorded. The first gesture vocabulary (G_1_) consists of three gestures executed by two workers from the TV assembly sector. They grabbed an electronic card from a container, took a wire from another, connected them, and placed them on a TV chassis. The first gesture is grabbing the electronic card from a container (G_1,1_), the second consists of taking a wire from a second container (G_1,2_), and the third one involves connecting the electronic card and wire and placing them on the TV chassis (G_1,3_).

The second gesture vocabulary is composed of three gestures performed in the airplane assembly sector (G_2_). Two workers were recorded, one performing riveting with a pneumatic hammer and the other holding a bucking bar to counteract the incoming rivet. The first gesture in this second vocabulary is riveting with the pneumatic hammer (G_2,1_), while the second is preparing the pneumatic hammer and grabbing rivets (G_2,2_), and the third involves positioning the bucking bar to counteract the incoming rivet (G_2,3_).

The third gesture vocabulary contains five gestures performed by a glassblower when creating a water carafe (G_3_). In the first gesture, the glassblower with a blowpipe grabs melted glass from an oven while rotating the blowpipe (G_3,1_). For the second gesture, the glassblower holds a specific paper with his right hand and shapes the carafe’s curves while seated in a metallic structure (G_3,2_). The third gesture involves blowing through the metal blowpipe that holds the glass for the carafe (G_3,3_). In the fourth gesture, the glassblower is shaping the carafe’s neck with pliers while standing (G_3,4_), and the fifth gesture concerns heating the glass of the carafe in the oven while rotating the blowpipe (G_3,5_).

The last gestural vocabulary is related to 28 motion primitives performed in a laboratory (G_4_), recorded from ten subjects. Each gesture exposed the subjects to a different level of ergonomic risk. According to EAWS, the ergonomic risk level depends on the torso, legs, and arms posture. The gestures here varied in the posture of the spine, legs, and arms. For the torso posture, in some gestures, the subjects bent more than $60^{\circ }$, rotated the torso, laterally bent to the left, or rotated their torso while leaning forward. The legs posture changes depending on whether the subject executes the gesture standing, sitting, or kneeling. Regarding the arms posture, the changes consist of raising their arms above shoulder level or keeping them down and having the arms stretched or bent $90^{\circ }$.
